# Exploring levels and factors associated with transition challenges for Syrian refugee parents resettled in Canada

**DOI:** 10.1371/journal.pone.0336392

**Published:** 2025-11-06

**Authors:** Hala Tamim, Elena Levitskaya, MacGregor Goodman, Gwen Ehi, Aliza Maqsood, Safoura Zangiabadi, Yunis Khaled

**Affiliations:** 1 Department of Kinesiology and Health Science, York University, Toronto, Ontario, Canada; 2 American University of Beirut, Beirut, Lebanon; Ankara University: Ankara Universitesi, TÜRKIYE

## Abstract

Although the literature has documented numerous challenges Syrian refugees face during their resettlement in Canada, the unique transition experiences of Syrian refugee parents remain underexplored. This study examines demographic, community and social, migration, and health-related factors that influence the level of difficulty experienced by Syrian refugee parents in Canada during their transition. This cross-sectional, interview-based study was conducted from March 2021 to March 2022, involving 540 Syrian refugee parents in Ontario with at least one child under the age of 18. Transition difficulty was measured based on the question “How difficult has the transition into Canada been for you?” Responses ranged from 1 representing “Not difficult at all” and 5 representing “Very difficult”. 6.5% of participants rated their transition as “Not difficult at all”, 15.9% as “Not difficult, “20.6% as “Neutral”, 43% as “Difficult”, and 13.7% as “Very difficult”. Results of the multiple linear regression analyses indicated that greater transition difficulty was significantly associated with experiences of discrimination at children’s school events (Adjβ = 0.138, p = 0.038), dissatisfaction with friendships (Adjβ = 0.134, p = 0.006), being over age 45 (Adjβ = 0.301, p = 0.047), lower proficiency in English or French (Adjβ = − 0.145, p = 0.008), longer duration spent in Canada (Adjβ = 0.123, p < 0.001), Blended Visa Office-Referred program (Adjβ = 0.530, p = 0.026) and poorer mental health (Adjβ = 0.173, p < 0.001). The findings from this study highlight the need for policies and frameworks aimed at improving resettlement efforts for refugee parents, thereby promoting the overall well-being of Syrian refugee families in Canada.

## Introduction

Since the outbreak of the Syrian civil war in 2011, tens of thousands of Syrian refugees have sought resettlement in various countries, including Canada [1]. Canada has played a pivotal role as a major host country, welcoming over 54,000 Syrian refugees as of February 29, 2024 [2]. This effort was facilitated through three main immigration streams: the Government-Assisted Refugees (GAR) program, which supports refugees identified by the United Nations High Commissioner for Refugees (UNHCR); the Privately Sponsored Refugees (PSR) program where private citizen groups assume full financial and emotional support for one year; the Blended Visa Office-Referred (BVOR) initiative, which shares sponsorship responsibilities between the government and private sponsors. Each of these programs offer a different framework of support and has implications for refugee integrations. In addition, Canada has offered comprehensive support, addressing basic needs, healthcare, language training, and social integration for refugees transitioning into Canadian society [1].

Despite these initiatives, the transition process remains fraught with multifaceted challenges that refugees face during resettlement, particularly in navigating the host country’s systems and culture. A growing body of research identifies persistent challenges in areas such as employment, housing, language acquisition, access to services, racism, and discriminations, as well as mental health. Language barriers significantly hinder access to healthcare, housing, education, and employment, limiting opportunities for social and economic integration [3,4]. In countries like Lebanon, Jordan, and Turkey, Syrian refugees often face underemployment due to non-recognition of qualifications and limited vocational training opportunities, compounded by language difficulties [5]. Affordable housing remains scarce, especially in high-demand urban areas like Toronto, where refugees settle, leading to overcrowded living conditions and housing insecurity, which further exacerbates instability, while cultural adaptation challenges, such as unfamiliar norms and xenophobia, foster alienation [6]. These barriers are mirrored globally, with refugees in the United States and Turkey encountering similar difficulties, highlighting the universal nature of integration challenges despite varying resettlement contexts [4,7]. Trauma from war and displacement frequently results in mental health challenges, complicating integration efforts, particularly in contexts where trauma-sensitive care is lacking [8].

Adjusting to a new social, cultural, and linguistic environment is a complex process, particularly for parents who must also ensure the well-being of their children during this transition [9]. Like other refugee parents, Syrian refugee parents serve as emotional and economic anchors for their families. This dual responsibility amplifies the pressures they face in adapting to a new society and navigating systemic barriers such as language, employment, and housing instability. These challenges not only impact parents’ mental health but also influence family dynamics and the developmental outcomes of their children during resettlement [10]. As primary caregivers and cultural mediators, parents play a central role in fostering their families’ successful adaptation to a new society [11].

While previous research has often considered Syrian refugees as a monolithic group, this study focuses specifically on Syrian refugee parents, whose experiences are uniquely shaped by their caregiving roles. Parents often bear dual responsibility of supporting their families emotionally and financially while simultaneously adapting to new cultural, linguistic, and institutional systems. Unlike non-parents, refugee parents must also navigate childcare, education systems, and intergenerational cultural shifts, adding layers of complexity to their integration journey. Their well-being directly impacts the developmental and integration of their children, making their successful adaptation critical not just at the individual level but for family and community resilience as a whole.

To analyze these experiences, this study draws upon two complementary theoretical frameworks: Berry’s Acculturation Model (1980) [12] and the Ager and Strang Integration Framework (2008) [13]. Berry’s model emphasizes the importance of both cultural maintenance and participation in the host society and categorizes acculturation strategies into four types: assimilation, separation, integration, and marginalization. These concepts provide a foundation for examining how refugee parents negotiatecultural identity and belonging during resettlement. This theoretical lens guided the development of the study objectives by shaping the understanding of how these cultural processes influence adaptiation and integration experiences. Ager and Strang’s framework offered a multidimensional perspective on refugee integration by identifying ten core domains organized into four overarching categories: markers and means (including employment, housing, education, and health), social connections (encompassing social bridges, bonds, and links); facilitators (such as language and cultural knowledge, as well as safety and stability); and foundations (including rights and citizenship). This framework guided the identification of key variables and informed the analytical structure, ensuring that both structural and social dimensions of integration were examined. Together, these frameworks provide a comprehensive understanding of refugee resettlement, capturing both systemic and relational aspects of integration and offering a robust analytical foundation for examining the transition experiences of Syrian refugee parents in Canada. Based on these theoretical frameworks, the study hypothesized that multiple interrelated factors, including social, linguistic, and systemic barriers, intersect to influence the level of transition difficulty experienced by Syrian refugee parents in Canada.

Throughout this study, the term “*transition difficulty”* is used to describe the complex, multi-layered experiences that Syrian refugee parents encounter while trying to adapt to life in Canada. This concept refers to the challenges associated with resettlement, encompassing practical aspects such as navigating housing, employment, healthcare, and language, as well as various emotional and psychological dimensions. These include experiences of uncertainty, parenting stress, social isolation, cultural dislocation, and the emotional toll of displacement and loss.

Since 2015, nearly half of all Syrian refugees in Canada, have settled in Ontario [14], and many of them are parents. Yet, there is limited research focused on the specific challenges faced by Syrian refugee parents. Thus, the aim of this study is to examine how demographic, community and social, migration, and health-related factors collectively influence the transition difficulties faced by Syrian refugee parents in Canada. Specifically, the study addresses two research questions: 1) What factors are associated with increased transition difficulty, and 2) How do these factors interact to shape the resettlement experience. The findings from this study provide valuable insights into how targeted interventions can ease transition difficulties for refugee families and improve their integration outcomes. Ultimately, improving the integration not only enhances the well-being of refugee families but also supports their long-term social and economic inclusion of refugee families in the Canadian society.

## Materials and methods

### Study participants

A cross-sectional, interview-based study was conducted from March 2021 to March 2022. Eligible participants were Syrian refugee parents residing in Ontario who had resettled in Canada after 2015 and had at least one child under the age of 18 at the time of the interview. Non-parents were excluded, as the study focused on resettlement experiences within the parenting context. The sample comprised of 540 individuals who met the inclusion criteria and were available during the data collection period.

Participants were recruited through convenience sampling, facilitated by partnerships with local service organizations, including Access Alliance Multicultural Health and the Arab Community Centre of Toronto. The survey was part of a broader community-based project examining the integration experiences of Syrian refugees in Canada, which captured demographic, migration, and health-related factors that influence resettlement and adaptation.

### Ethical considerations and data collection

The project was approved by the Research Ethics Board at York University (Certificate # e2019-128). In adherence to COVID-19 social distancing protocols [15], participant recruitment and data collection were conducted via telephone. Prior to survey administration, Research Assistants provided participants with a digital copy of the consent form, which outlined the study objectives, voluntary nature of participation, and the option to withdraw at any time without penalty. Research Assistants reviewed the consent form with each participant, addressing any questions or concerns. The survey was then administered by research assistants fluent in Arabic, especially the Syrian dialect. Participant responses were recorded using Qualtrics, a secure password-protected online data collection platform. To compensate participants for their time and involvement, a $20 honorarium was provided. Additionally, participants who reported poor self-rated mental health were referred to appropriate local mental health agencies for support, in accordance with the study protocol.

### Assessment of transition challenges, socio-demographic characteristics, community and social support, migration experiences, and health-related factors

Information about transition difficulties was collected based on the question “How difficult has the transition into Canada been for you?” The answers ranged from 1 representing “Not difficult at all” to 5 representing “Very difficult”. The demographic characteristics considered for the study included gender (being a mother/ father), age (categorized as ≤35/ 35–44/ ≥ 45), number of children, highest level of education as a continuous variable on a scale ranging from 1 to 10, from no formal education to professional degree, respectively. Language proficiency in English or French was assessed by the question “Please rate your current overall ability in English or French, whichever you are more comfortable with” ranged from 1 representing “Not at all” to 6 representing “Excellent”, working status (yes/ no), and religion (Muslim/ Non-Muslim). The migration-related factors included the type of sponsorship involved in the process of becoming a refugee (GAR/ PSR/ BVOR) and number of years in Canada. Health-related factors included information on self-rated mental health collected on a 5-point Likert scale, with 1 representing an “Excellent” rating and 5 representing a “Poor” rating. Lastly, the community and social factors involved housing satisfaction, discrimination in school events, and social support. Housing satisfaction information was collected based on the question “Overall, how satisfied are you with your housing?” The answers ranged from 1 representing “Very satisfied” to 5 representing “Very unsatisfied”. Discrimination in school events was assessed by the question “How often do you as a parent face discrimination in your child(ren)’s school events?” The answers ranged from 1 representing “Never” to 5 representing “Always.” Also, social support was measured as satisfaction with friends from the question “How satisfied are you with the quality of the friendships you have here?” Answers were scaled from 1 to 5, from “Very satisfied” to “Very unsatisfied”, respectively.

### Statistical analyses

Simple linear regression models were performed to assess the bivariate relationship between each of the demographic, community, social, migration, and health-related factors with transition difficulties. In addition, a multiple linear regression model was conducted for the outcome variable. The beta coefficient and 95% confidence intervals (95% CIs) were reported. All regression models were adjusted for the clustering effect of belonging to the same family. All analyses were conducted using the Statistical Package for the Social Sciences (SPSS, version 26.0).

## Results

The transition difficulties of Syrian refugee parents in Canada were assessed based on the percentage distribution of their perceived level of difficulty during the transition process. The majority of the respondents (43%) reported their transition as “Difficult”, while 13.7% reported it as “Very Difficult”. In contrast, fewer respondents (15.9%) indicated lower levels of difficulty, with 15.9% reporting it as “Not Difficult” and 6.5% as “Not difficult at all”.

Table 1 presents the descriptive statistics for the characteristics of study participants and the bivariate relationships between Syrian refugee parents’ difficulties in transitioning to Canada and factors related to demographic characteristics, community and social support, migration experiences and health. A total of 540 participants were included in this study, with the majority being mothers (60.6%) and an average of 3.4 children per participant (SD = 1.5). Nearly half of the participants (47.2%) were within the 36–44 age category and had been living in Canada for an average of 3.90 years (SD = 1.50). The participants’ average education level was 6.74 (SD = 2.46) on a scale ranging from 1 (no formal education) to 10 (professional degree), with 65.6% being unemployed. Additionally, 57.8% of participants had a private method of sponsorship, and their average self-rated ability to speak English or French was 3.97 (SD = 1.23) on a scale of 1 (not at all) to 6 (excellent).

**Table 1:** Descriptive statistics and bivariate relationships between syrian refugee parents’ difficulty in transitioning to canada and variables of community and social factors, socio-demographic factors, migration-related factors, and health-related factors.

Factor	Number	(%)	Mean (SD)	Difficulty of transition for parents		
				Unadjusted β (SE)	p-value	95% CI
Demographic characteristics						
Gender						
Mother	329	60.9		ref		
Father	211	39.1		0.026 (0.097)	0.792	−0.165, 0.216
Age						
≤35	154	28.5		ref		
36-44	255	47.2		0.159 (0.113)	0.162	−0.064, 0.382
≥45	131	24.3		0.319 (0.133)	0.017*	0.058, 0.579
Number of Children			3.35 (1.47)	0.078 (0.033)	0.017*	0.014, 0.142
Education^			6.74 (2.46)	−0.081 (0.020)	<0.001**	−0.121, −0.042
Language proficiency in English or French^#^			3.97 (1.23)	−0.211 (0.037)	<0.001**	−0.284, −0.137
Working status						
Yes	186	34.4		−0.222 (0.100)	0.027*	−0.418, −0.025
No	354	65.6		ref		
Religion						
Muslim	340	63.0		0.303 (0.104)	0.004**	0.099, 0.507
Non-Muslim	192	35.6		ref		
Community and Social factors						
Housing Satisfaction†			2.74 (1.16)	0.108 (0.041)	0.009**	0.028, 0.189
Discrimination in School Events‡			1.27 (0.73)	0.193 (0.067)	0.004**	0.061, 0.324
Satisfaction with friends†			2.52 (1.06)	0.179 (0.045)	<0.001**	0.091, 0.266
Migration- and Health- related factors						
Sponsorship						
Governmental	202	37.4		ref		
Private	312	57.8		−0.226 (0.101)	0.025*	−0.424, −0.029
Other	26	4.8		0.374 (0.228)	0.101	−0.073, 0.822
Number of years in Canada			3.90 (1.57)	0.030 (0.033)	0.351	−0.034, 0.094
Self-rated Mental Health***			3.06 (1.19)	0.274 (0.038)	<0.001**	0.199, 0.349

†Scale from 1-5 (1=Very satisfied and 5=Very unsatisfied)

‡Scale from 1-4 (1=Never and 5=Very often)

^ Scale from 1-10 (1=No formal education, 10=professional degree)

#Scale from 1-6 (1=Not at all, 6=Excellent)

***Scale from 1-5 (1=Excellent and 5=Poor)

*p < 0.05

**p < 0.01

At the bivariate level, significant associations were identified between parents’ challenges in transitioning to Canada and various factors, including socio-demographic characteristics, community and social support, migration and health-related factors. Among socio-demographic characteristics, older age (≥45 years) was significantly associated with greater transition difficulty compared to those aged ≤35 (UAdj β = 0.319, p = 0.017). Similarly, having more children was positively associated with transition difficulty (UAdj β = 0.078, p = 0.017). Conversely, higher education levels,proficiency in English or French, and being employed were associated with lower transitiondifficulty (UAdj β = −0.081, p < 0.001; UAdj β = −0.211, p < 0.001; UAdj β = −0.222, p = 0.027). Being Muslim was significantly associated with greater transition difficulty (Uadj β = 0.303, p = 0.004). Of community and social factors, greater dissatisfaction with housing (UAdj β = 0.108, p = 0.009), more experiences of discrimination in school events (UAdj β = 0.193, p = 0.004), and lower satisfaction with friendships (UAdj β = 0.179, p < 0.001) were all significantly associated with greater difficulty. With respect to migration-related factors, privately sponsored parents reported significantly lower difficulty compared to government-sponsored parents (UAdj β = −0.226, p = 0.025). Poorer self-rated mental health was also significantly associated with greater transition difficulty (UAdj β = 0.274, p < 0.001).

Table 2 summarizes the results of the multiple linear regression analysis, with an overall R-squared value of 0.192. After adjusting for all other variables, participants who reported lower satisfaction with their quality of friendships experienced greater difficulty transitioning to Canada (Adjβ = 0.134, p = 0.006). Similarly, parents who faced discrimination at their children’s school events encountered more challenges during their transition in Canada (Adjβ = 0.138, p = 0.038). Moreover, parents with lower levels of proficiency in English and French exhibited significantly higher levels of difficulty transitioning (Adjβ = − 0.145, p = 0.008). The age of parents was also significantly associated with the level of difficulty in transitioning, with parents aged 45 years or older experiencing significantly greater challenges in their transition compared to younger parents (Adjβ = 0.301, p = 0.047). Results revealed that the method of sponsorship to Canada was significantly associated with transition difficulty. Parents sponsored through a BVOR program reported greater difficulty in transitioning compared to those sponsored through government sponsorship programs (Adjβ = 0.530, p = 0.026). Additionally, the number of years spent in Canada was positively associated with transition difficulty, with longer residency correlating with greater challenges. Lastly, self-rated mental health was strongly associated with transition difficulty. Parents with poorer self-rated mental health reported significantly higher levels of difficulty transitioning (Adjβ = 0.173, p < 0.001).

**Table 2:** Results of the multivariate regression analysis on factors associated with syrian refugee parents’ difficulty in transitioning to canada, including community and social support, socio-demographic factors, migration-related factors, and health-related factors.

Factor	Difficulty of transition for parents		
	Adjusted β (SE)	p-value	95% CI
Demographic characteristics			
Gender			
Mother	ref		
Father	−0.016 (0.113)	0.884	−0.238, 0.205
Age			
≤35	ref		
36-44	0.185 (0.123)	0.132	−0.056, 0.427
≥45	0.301 (0.151)	0.047*	0.004, 0.597
Number of Children	−0.026 (0.042)	0.537	−0.109, 0.057
Education ^	−0.041 (0.028)	0.148	−0.096, 0.015
Language proficiency in English or French^#^	−0.145 (0.055)	0.008**	−0.253, −0.037
Working status			
Yes	−0.045	0.709	−.279, 0.190
No	ref		
Religion			
Muslim	0.182 (0.135)	0.176	−0.082, 0.447
Non-Muslim	ref		
Community and social factors			
Housing Satisfaction†	0.017 (0.044)	0.702	−0.069, 0.103
Discrimination in School Events‡	0.138 (0.066)	0.038*	0.007, 0.268
Satisfaction with friends†	0.134 (0.048)	0.006**	0.039, 0.228
Migration- and Health- related factors			
Sponsorship			
Governmental	ref		
Private	0.079 (0.135)	0.558	−0.186, 0.344
Other	0.530 (0.238)	0.026*	0.063, 0.996
Number of years in Canada	0.123 (0.037)	0.001**	0.050, 0.196
Mental Health***	0.173 (0.043)	<0.001**	0.088, 0.257

†Scale from 1-5 (1=Very satisfied and 5=Very unsatisfied)

‡Scale from 1-4 (1=Never and 5=Very often)

^ Scale from 1-10 (1=No formal education, 10=professional degree)

#Scale from 1-6 (1=Not at all, 6=Excellent)

***Scale from 1-5 (1=Excellent and 5=Poor)

*p < 0.05**p < 0.01

## Discussion

The objective of this study was to examine how demographic, community and social, migration, and health-related factors experiences shape the resettlement of Syrian refugee parents in Canada. The results of this study underscore key factors which impact the level of difficulty Syrian refugee parents experience as they transition into life in Canada. Experiences of discrimination at school events and dissatisfaction with friendships are both associated with a more difficult reported transition. Refugee parents who were not yet proficient in French or English, and parents over the age of 45 also reported greater difficulty. The results also indicated that refugees with blended sponsorship reported more significant difficulty adjusting to life in Canada. The number of years spent in Canada was also found to be positively associated with levels of difficulty, meaning, more time spent in Canada led participants to perceive the transition to be more difficult. Finally, poor self-reported mental health was associated with greater reported difficulty. The identification of these factors can guide targeted intervention to increase the ease with which refugee parents may transition into their new life in Canada. Overall, the findings confirm that multiple interrelated factors contribute to the challenges experienced during resettlement, supporting the study’s hypothesis that social, linguistic, and systemic barriers intersect to complicate integration processes.

The study found that school discrimination and social support were key community and social factors influencing transition difficulties for Syrian refugee parents in Canada. These findings are consistent with previous research, which found that Syrian refugee parents who faced discrimination at their children’s school events encountered significantly more challenges during their transition, hindering their ability to engage with the school community [16]. Another study found that discrimination also exacerbated existing trauma symptoms, negatively impacting the psychological well-being and academic achievement of Syrian refugee children. This, in turn, impacted parents’ ability to support and engage with the school system effectively, creating additional barriers to their overall transition and integration into Canadian society [17]. Indeed, refugee children’s well-being has been found to be significantly impacted by strong interpersonal relationships between members of the family and within the community in which they live [18].

Additionally, the study found that lower satisfaction with friendships further worsened transition challenges for Syrian refugee parents in Canada. This is consistent with previous research, which has highlighted the crucial role of social support in the adaptation process for refugees, with lower satisfaction leading to greater isolation and stress [19,20]. In these studies, high levels of resettlement stressors were associated with greater psychological distress, and the lack of social support intensified these challenges. Other research has identified systemic issues such as limited resources and narrow service mandates as hinderances to newcomers’ access to necessary support, further increasing isolation and stress [21].

With regard to demographic characteristics, both age and proficiency in English or French were significant factors associated with greater difficulty adapting to life in Canada. Parents over the age of 45 reported greater levels of difficulty compared to younger participants. This is consistent with previous research, which highlighted unique challenges that older Syrian refugees (over the age of 50) faced in their resettlement in Canada [22]. There is a need for further research to identify specific factors contributing to older Syrian refugee parents’ more difficult transition experience. Language proficiency in English or French was associated with lower levels of difficulty transitioning to life in Canada. This is consistent with previous research; for example, Syrian refugee mothers in Toronto with limited proficiency in English described feelings of fear and a loss of personal agency in matters related to their own health and the well-being of their children [23]. Interestingly, a study found that Syrian refugee parents in Canada were more likely to endorse speaking exclusively in their first language at home compared to Syrian refugee parents in Germany [24]. The dual importance of maintaining refugees’ first language and acquiring a new language has been widely recognized as essential for promoting positive psychological and social outcomes among migrants [25]. This finding aligns with Berry’s Acculturation Model (1980) [12], which highlights the need to balance cultural maintenance and participation in the host society to achieve successful integration. Therefore, it is crucial to reduce language barriers and create opportunities beyond the home for Syrian refugee parents to improve their proficiency in English or French.

The study found that both sponsorship type and length of time in Canada significantly influenced transition difficulties for Syrian refugee parents, with those resettled through the Blended Visa Office-Referred (BVOR) program facing more complex transitions than their counterparts in the Government-Assisted Refugees (GAR) stream. Although BVOR refugees accessed language assessments at similar rates, they were less likely to participate in follow-up language training, which hindered their integration and autonomy [19,26]. This discrepancy reflects structural weaknesses in the BVOR model, which pairs private sponsorship with reduced government support, often without sufficient oversight or sponsor preparedness. Many BVOR sponsors receive limited training or guidance, which constrains their ability to help refugee families navigate settlement services, find employment, or foster cultural adaptation [27]. Research by Ali-Hassan et al. (2021) found that BVOR parents reported higher levels of stress than GARs and Privately Sponsored Refugees (PSRs), due in part to inconsistent sponsor involvement and greater financial insecurity [28]. Immigration, Refugees and Citizenship Canada (IRCC)’s own evaluations support this, showing that BVOR refugees accessed fewer employment services and were less likely to have stable housing or income within their first year [27]. These structural gaps highlight the need for targeted reform. Strengthening the BVOR program requires mandatory sponsor training, regular follow-up from settlement agencies, and clearer accountability mechanisms to ensure sustained engagement. As Sim et al. (2023) emphasize, the mental health and coping capacity of refugee parents are tightly linked to the quality and continuity of post-arrival support [19]. Furthermore, the relative invisibility of BVOR-sponsored parents in existing research highlights the urgent need for more targeted data collection and evaluation. Improving support for this population will not only enhance parent-level outcomes but also indirectly benefit the family systems in which they play a central role.

The study also showed that Syrian refugee parents who had lived in Canada longer perceived the transition as more difficult. While counterintuitive, this finding aligns with research indicating that psychological distress among refugees can increase over time, particularly among those who arrived during earlier phases of resettlement [29]. One possible explanation lies in the structure of Canadian settlement support, which is typically concentrated in the first 12–24 months following arrival. During this period, refugees receive financial assistance, language training, and employment services through programs such as the Resettlement Assistance Program (RAP). However, these supports diminish or end entirely after the first two years, leaving many refugees to navigate ongoing challenges, such as housing instability, employment precarity, and health service access on their own [27]. Additionally, long-term integration efforts often overlook the continued needs of refugee families, particularly in areas like culturally competent mental health care, parenting supports, and elder-specific services [14,20]. This gap can be especially burdensome for refugee parents, who must manage both their own adaptation and that of their children. Sim et al. (2023) note that settlement-related stressors often accumulate over time and can become more difficult to manage without sustained support or social networks [19]. Moreover, Ghahari et al. (2020) found that structural barriers, such as narrow service mandates, language limitations, and service fragmentation persist beyond the initial resettlement phase, contributing to increased isolation and unaddressed needs among long-term refugees [20]. Thus, while early months in Canada may be marked by optimism and intensive support, the longer-term transition can become more challenging as institutional assistance wanes and barriers persist. These findings suggest the importance of extending integration services beyond the initial resettlement phase and tailoring them to evolving needs, particularly for parents and older refugees who may face compounded vulnerabilities over time.

Mental health was the only health-related factor that was measured and found to be associated with greater difficulty transitioning into life in Canada. Syrian refugee parents who reported poorer mental health also reported facing greater challenges during their resettlement into Canada. Previous research has highlighted Syrian refugees’ increased vulnerability experiencing poor mental health [30,31]. Mental health challenges among Syrian refugees more broadly may be attributed to experiences of severe trauma prior to migration [32] but may also be attributed to or compounded by transitional difficulty. Research has found that refugees in Canada underutilize mental health services, and one study identified that Syrian refugee mothers express apprehension about accessing services due to fears surrounding the involvement of child protective services [33]. Such findings further highlight the importance of accessible and culturally appropriate services for Syrian refugee parents, as well as the importance of advocacy and education as they navigate these services.

This study is the first to examine the transition challenges experienced by Syrian refugee parents in Canada. However, there are several limitations that need to be considered when interpreting the findings. First, the cross-sectional design precludes any causal interpretations of the observed associations. While we examined relationships in one direction, such as self-rated mental health predicting difficulty in resettling, it is also plausible that the reciprocal relationships exist. Longitudinal studies would be better suited to capture how refugee adjustment evolves over time and to clarify temporal relationships among key variables. Second, the use of self-reported measures introduces potential information bias, including recall and social desirability bias. While self-reporting offers important insights into lived experiences, it may not fully capture the complexity or accuracy of service access or psychological distress. Future studies could strengthen validity by triangulating parent-reported data with administrative records or service provider reports, such as those from settlement agencies, schools, or healthcare providers. Third, there is a risk of selection bias due to the use of convenience sampling. Fourth, the survey was developed for this study in collaboration with community stakeholders, ensuring contextual relevance but potentially limiting the generalizability of findings to other populations. Lastly, the results may also be influenced by confounding biases, such as differences in personality traits like coping abilities or resilience in the face of difficulty, which were not accounted for in this study.

## Conclusion

This study explored various factors influencing the level of difficulty Syrian refugee parents encounter during their transition to Canada, including demographic-, community and social, migration-, and health-related factors. The findings revealed that individuals who experienced discrimination at their children’s school event and those dissatisfied with their friendship reported higher levels of difficulty in their transition. Additionally, older parents (aged 45 and above) and those with limited English or French language proficiency encountered greater transition difficulty. Similarly, individuals sponsored through a BVOR program and those with poorer mental health reported increased difficulty in their transition experience. These results highlight the critical need for language interpretation services, culturally inclusive social initiatives, and enhancements to sponsorship programs to support refugee families more effectively. Providing refugee parents with adequate resources can improve their ability to navigate the transition process more strategically and better support their children, ultimately fostering positive family outcomes. Future research should evaluate the effectiveness of existing support services and incorporate a broader range of psychosocial and structural variables to better capture the complexity of refugee resettlement experiences.
